# Entropy Power, Autoregressive Models, and Mutual Information

**DOI:** 10.3390/e20100750

**Published:** 2018-09-30

**Authors:** Jerry Gibson

**Affiliations:** Department of Electrical and Computer Engineering, University of California, Santa Barbara, Santa Barbara, CA 93106, USA; gibson@ece.ucsb.edu

**Keywords:** autoregressive models, entropy rate power, mutual information

## Abstract

Autoregressive processes play a major role in speech processing (linear prediction), seismic signal processing, biological signal processing, and many other applications. We consider the quantity defined by Shannon in 1948, the entropy rate power, and show that the log ratio of entropy powers equals the difference in the differential entropy of the two processes. Furthermore, we use the log ratio of entropy powers to analyze the change in mutual information as the model order is increased for autoregressive processes. We examine when we can substitute the minimum mean squared prediction error for the entropy power in the log ratio of entropy powers, thus greatly simplifying the calculations to obtain the differential entropy and the change in mutual information and therefore increasing the utility of the approach. Applications to speech processing and coding are given and potential applications to seismic signal processing, EEG classification, and ECG classification are described.

## 1. Introduction

In time series analysis, the autoregressive (AR) model, also called the linear prediction model, has received considerable attention, with a host of results on fitting AR models, AR model prediction performance, and decision-making using time series analysis based on AR models [1,2,3]. The linear prediction or AR model also plays a major role in many digital signal processing applications, such as linear prediction modeling of speech signals [4,5,6], geophysical exploration [7,8], electrocardiogram (ECG) classification [9], and electroencephalogram (EEG) classification [10,11]. We take a new look at AR modeling and analysis and these applications using a quantity from the field of information theory as defined by Shannon.

In his landmark 1948 paper [12], Shannon defined what he called the *derived quantity* of entropy power (also called entropy rate power) to be the power in a Gaussian white noise limited to the same band as the original ensemble and having the same entropy. He then used the entropy power in bounding the capacity of certain channels and for specifying a lower bound on the rate distortion function of a source. Entropy rate power appears to be as fundamental a concept in Shannon Information Theory as mutual information (then called average mutual information [13]); however, unlike mutual information and many other quantities in Shannon theory, entropy rate power is not a well-studied, widely employed concept, particularly in signal processing.

We examine entropy rate power from several different perspectives that reveal new possibilities, and we define a new quantity, the log ratio of entropy powers, that serves as a new tool in the information theoretic study of a sequence of signal processing operations. In particular, we use the log ratio of entropy powers to study the change in differential entropy and the change in mutual information as the predictor order is increased for autoregressive processes. We also demonstrate the use of the log ratio of entropy powers for the analysis of speech waveform coders and the analysis of code-excited linear predictive coding of speech. Specifically, for speech waveform coding the overall reduction in mean squared prediction error can be interpreted using the log ratio of entropy powers as the performance gain in terms of bits/sample provided by the predictor. This insight is entirely new. For the analysis of code-excited linear predictive speech coding, the log ratio of entropy powers allows us to decompose the mutual information between the input speech being coded and the reconstructed speech into a sum of mutual informations and conditional mutual informations corresponding to the information provided by each of the components of the code-excited speech codec. This allows us to characterize the contributions of each component in terms of the mutual information with respect to the input speech. This is an entirely new way of analyzing the performance of code-excited linear predictive coding and potentially offers new design approaches for this dominant speech coding structure.

Additionally, we consider the applications of the AR model in geophysical exploration [7,8], electrocardiogram (ECG) classification [9], and electroencephalogram (EEG) classification [10,11] and setup how the log ratio of entropy powers can provide new insights and results for these fields. For each of these applications, the interpretation of change in mean squared prediction error for different predictor orders in terms of changes in differential entropy and changes in mutual information open up new analysis and classification paradigms. Separately, Gibson and Mahadevan [14] have used the log ratio of entropy powers to derive and extend the interpretation of the log likelihood spectral distance measure from signal processing.

We begin by defining the concept of entropy power and entropy rate power in Section 2 and there summarize the well known results for entropy power in terms of the power spectral density of a Gaussian process. Section 3 presents the autoregressive model and power spectral density and the Levinson-Durbin recursion for the solution of the Toeplitz set of equations that occurs when solving for the AR or linear prediction coefficients of an asymptotically wide-sense stationary AR process as delineated in [6,15,16]. The equivalence of the entropy power and the minimum mean squared one step ahead prediction error for Gaussian AR sequences and relevant bounds on the minimum mean squared prediction error for non-Gaussian processes are discussed in Section 4.

The fundamental new quantity, the log ratio of entropy powers, is developed in Section 5, where it is shown that the key results hold when the entropy power is replaced by the variance for Laplacian and Gaussian processes and where the relationship of the log ratio of entropy powers to changes in differential entropy and mutual information is presented as a signal progresses through a series of linear predictors when the model order is incremented. Also in this section, the log ratio of entropy powers for maximum entropy spectral estimates and for decompositions in terms of orthogonal deterministic and nondeterministic processes are examined.

Examples are presented in Section 6 illustrating the use of the log ratio of entropy powers for the linear prediction analysis of several speech frames as the predictor order is increased. Applications to speech waveform coding and to speech coding using code-excited linear prediction are given in Section 7. Other possible applications to AR modeling in geophysical exploration [7,8], ECG classification [9], and EEG classification [10,11] are outlined in Section 8. Conclusions and suggestions for future applications are provided in Section 9.

## 2. Entropy Power/Entropy Rate Power

In his landmark 1948 paper [12], Shannon defined the entropy power (also called entropy rate power) to be the power in a Gaussian white noise limited to the same band as the original ensemble and having the same entropy. He then used the entropy power in bounding the capacity of certain channels and for specifying a lower bound on the rate distortion function of a source. We develop the basic definitions and relationships concerning entropy power in this section for use later in the paper.

Given a random variable *X* with probability density function p(x), we can write the differential entropy

(1) $$h\left(X\right)=-\int_{-\infty }^{\infty }p(x)\log p(x)dx$$

where *X* has the variance var(X) = $\sigma^{2}$. Since the Gaussian distribution has the maximum differential entropy of any distribution with mean zero and variance $\sigma^{2}$ [17],

(2) $$h\left(X\right)\leq \frac{1}{2}\log2\pi e\sigma^{2}$$

from which we obtain

(3) $$Q=\frac{1}{\left(2\pi e\right)}\exp2h(X)\leq \sigma^{2}$$

where *Q* was defined by Shannon to be the *entropy power* associated with the differential entropy of the original random variable *X* [12]. In addition to defining entropy power, this equation shows that the entropy power is the *minimum variance* that can be associated with the not-necessarily Gaussian differential entropy h(X).

Please note that Equation (3) allows us to calculate the entropy power associated with any given entropy. For example, if the random variable *X* is Laplacian [18] with parameter λ, then h(X)=ln(2λe) and we can substitute this into Equation (3) and solve for the entropy power $Q=2e\lambda^{2}/\pi$. Since the variance of the Laplacian distribution is $\sigma^{2}=2\lambda^{2}$, we see that $Q<\sigma^{2}$, as expected from Equation (3). This emphasizes the fact that the entropy power is not limited to Gaussian processes. This simple result is useful since speech signals as well as the linear prediction error for speech are often modeled by Laplacian distributions.

For an *n*-vector X with probability density $p(x^{n})$, and covariance matrix $K_{X}=E\left[\left(X-E\left(X\right)\right){\left(X-E\left(X\right)\right)}^{T}\right]$, we have that

(4) $$h\left(X\right)\leq \frac{1}{2}\log\left[{\left(2\pi e\right)}^{n}|K_{X}|\right]$$

from which we can construct the vector version of the entropy power as

(5) $$Q_{X}=\frac{1}{{\left(2\pi e\right)}^{n}}\exp2h(X)\leq \left|K_{X}\right|.$$

We can write a conditional version of Equation (3) as

(6) $$Q_{X|Y}=\frac{1}{\left(2\pi e\right)}\exp2h(X|Y)\leq Var\left(X|Y\right)$$

We will have the occasion to study pairs of random vectors X and Y where we use the vector Y to form the best estimate of X. If $K_{X|Y}$ is the covariance matrix of the minimum mean squared error estimate of X given Y, then we have

(7) $$h\left(X|Y\right)\leq \frac{1}{2}\log\left[{\left(2\pi e\right)}^{n}|K_{X|Y}|\right]$$

and from which we can get an expression for the conditional entropy power, $Q_{X|Y}$,

(8) $$Q_{X|Y}=\frac{1}{{\left(2\pi e\right)}^{n}}\exp2h(X|Y)\leq \left|K_{X|Y}\right|.$$

So, $Q_{X|Y}$ is upper bounded by the determinant of the conditional error covariance matrix, $|K_{X|Y}|$. We have equality in Equations (2)–(8) if the corresponding random variables or random vectors are Gaussian.

Often our interest is in investigating the properties of stationary random processes. Thus, if we let **X** be a stationary continuous-valued random process with samples $X^{n}$ =$\left[X_{i},i=1,2,\ldots ,n\right]$, then the differential entropy rate of the process **X** is [19]

(9) $$\bar{h}=\lim_{n\to \infty }\frac{1}{n}h(X^{n})=\lim_{n\to \infty }h(X_{n}|X^{n-1})$$

We assume that this limit exists in our developments and we drop the overbar notation and use *h* = $\bar{h}$. Using the entropy rate in the definition of entropy power yields the nomenclature *entropy rate power*.

Within the context of calculating and bounding the rate distortion function of a a discrete-time stationary random process, Kolmogorov [20] and Pinsker [21] derived an expression for the entropy rate power in terms of its power spectral density.

If we now consider a discrete-time stationary Gaussian process with correlation function ϕ(k) = $E[X_{j}X_{j+k}]$, the periodic discrete-time power spectral density is defined by

(10) $$\Phi \left(\omega \right)=\sum_{-\infty }^{\infty }\phi (k)\exp\left(j\omega k\right)$$

for |ω|≤π. We know that an *n*-dimensional Gaussian density with correlation matrix $\Phi_{n}$ has the differential entropy h(X) = $\left(n/2\right)\log\left(2\pi e|\Phi_{n}{|}^{1/n}\right)$. Then, the entropy rate power *Q* can be found from [22,23]

(11) $$\log Q=\lim_{n\to \infty }\log|\Phi_{n}{|}^{1/n}$$

which yields [22,23]

(12) $$Q=\exp[\frac{1}{2\pi }\int_{-\pi }^{\pi }\log\Phi (\omega )d\omega ]$$

as the entropy rate power.

Later we develop a closely related result for any distribution using the definition of entropy power.

## 3. Autoregressive Models

An AR process is given by

(13) $$s\left(k\right)=\sum_{i=1}^{m}a_{i}s\left(k-i\right)+w\left(k\right)$$

where the autoregressive parameters $a_{i},i=1,2,\ldots ,m$, are called the linear prediction coefficients for speech processing applications, and w(k) is the excitation sequence. For purposes of linear prediction speech analysis, we generally do not need to make an assumption on the distribution of the excitation. However, it is often assumed that the prediction error for speech is Laplacianly distributed [6,18,24].

In general time series analysis, the excitation is often chosen to be some i.i.d. sequence, the distribution of which is unspecified. The AR model in Equation (13) is a one-sided process and depends on the initial conditions, which we will assume to be zero, and therefore is not wide sense stationary (WSS). As a result, the power spectral density of the AR process is not accurately represented by Equation (10). Fortunately, an important result due to Gray [15], provides the necessary power spectral density expression for AR processes.

### 3.1. The Power Spectral Density

For the AR model in Equation (13), the correlation function ϕ(k) = $E[X_{j}X_{j+k}]$ depends on *j* and therefore is not WSS. However, the AR model can be shown to be asymptotically WSS under suitable conditions [15,25,26,27]. Leaving the details to the references, for asymptotically WSS AR processes with coefficients $a_{i},i=0,1,2\ldots ,m$, the asymptotic power spectral density is given by

(14) $$\Phi_{AR}\left(\omega \right)=\frac{\sigma^{2}}{g(\omega )}$$

where

(15) $$g\left(\omega \right)=|\sum_{k=0}^{m}a_{k}{\exp\left(j\omega k\right)|}^{2}$$

with $\sigma^{2}$ the variance of the AR process input sequence and $a_{0}\equiv 1$.

$\Phi_{AR}\left(\omega \right)$ is the power spectral density of the AR process that we use in the remainder of the paper.

### 3.2. The Levinson-Durbin Recursion

In linear prediction for speech analysis and coding, the linear prediction model is used to capture the spectral envelope. For a given windowed frame of *L* input speech samples, where the windowing sets all samples outside the window to zero, it is necessary to calculate the linear prediction coefficients. This is done by choosing the coefficients to minimize the sum of the squared prediction errors over the frame; that is, choose the coefficients to minimize $\epsilon^{2}\left(k\right)=\sum {\left[s\left(k\right)-\sum_{i=1}^{m}a_{i}s\left(k-i\right)\right]}^{2}$ [4,5,6]. Taking the partial derivatives with respect to each of the coefficients, $a_{j}$, j=1,2,…,m, yields the set of linear simultaneous equations

(16) $$\sum_{i=1}^{m}a_{i}R(|i-j|)=R\left(j\right)$$

for j=1,2,…,m, where

(17) $$R\left(j\right)=\frac{1}{L}\sum_{k=0}^{L-|j|-1}s\left(k\right)s\left(k+|j|\right)$$

with R(j)=R(−j). In matrix notation this becomes

(18) RA=C

where **R** is an *m* by *m* Toeplitz matrix of the autocorrelation terms in Equation (17), $A={\left[a_{1},a_{2},\ldots ,a_{m}\right]}^{T}$, and C is a column vector of the autocorrelation terms R(j),j=1,2,…,m.

An efficient method to solve the set of linear simultaneous equations in Equation (18) is due to Durbin [28] and is given by [4,5,6,16]:

Let

(19) $$E^{\left(0\right)}=R\left(0\right)$$

then compute

(20) $$p_{n}=\left[R\left(n\right)-\sum_{j=1}^{n-1}a_{j}^{\left(n-1\right)}R\left(n-j\right)\right]/E^{\left(n-1\right)}$$

for 1≤n≤m. Then we compute the linear prediction coefficients as

(21) $$a_{n}^{\left(n\right)}=p_{n}$$

and

(22) $$a_{j}^{\left(n\right)}=a_{j}^{\left(n-1\right)}-p_{n}a_{n-j}^{\left(n-1\right)}.$$

We next calculate

(23) $$E^{\left(n\right)}=\left(1-p_{n}^{2}\right)E^{\left(n-1\right)}$$

and then go to Equation (20) and continue until n=m, the desired predictor order.

The quantity $E^{\left(n\right)}$ is the mean squared prediction error (MSPE) for the *n*th order predictor, which is also given by the expression

(24) $$E^{\left(n\right)}=R\left(0\right)-\sum_{j=1}^{n}a_{j}^{\left(n\right)}R\left(j\right).$$

and is nonincreasing with increasing predictor order so we have that

(25) $$R\left(0\right)=E^{\left(0\right)}\geq E^{\left(1\right)}\geq E^{\left(2\right)}\geq \cdots \geq E^{\left(m-1\right)}\geq E^{\left(m\right)}.$$

In many problems, such as speech coding applications, the predictor order *m* is chosen based on a combination of predictor accuracy over a wide set of speech data and a desired bit rate allocation to the set of coefficients.

However, for speech analysis, one can monitor the MSPE and set the predictor order to the value where further decreases in $E^{\left(n\right)}$ are small. For applications to seismic exploration [7], electrocardiogram (ECG) classification [9], and electroencephalogram (EEG) classification [10,11], the AR model order may be fixed to a preselected value, determined via the Durbin recursion according to an acceptable MSPE, or investigated and adjusted for each experimental application. We discuss each explicitly in Section 8.

Later we use the sequence of mean squared prediction errors in Equation (25) to infer the change in mutual information as the predictor order is increased, but to justify this approach, first we must develop the key concepts and expressions relating entropy power and minimum mean squared prediction error, which we do in the next section.

## 4. Minimum MSPE and AR Models

Perhaps surprisingly, the right side of Equation (12) plays a major role in time series analysis beyond entropy power and the Gaussian assumption. More specifically, using $\Phi_{AR}\left(\omega \right)$ in Equation (12) we obtain the minimum mean squared one-step prediction error (MSPE) for autoregressive (AR) processes, even if they are not Gaussian [2,3,22]! That is, the mean squared prediction error, sometimes called the innovation variance [3], is given by

(26) $$E^{\left(\infty \right)}=\exp[\frac{1}{2\pi }\int_{-\pi }^{\pi }\log\Phi_{AR}\left(\omega \right)d\omega ]$$

where we have used the notation from Section 3 for the minimum mean squared prediction error. This result does not require the AR process to be Gaussian.

Furthermore, the fact that Equation (26) is the minimum mean squared one-step prediction error for AR processes, even non-Gaussian processes, proves useful for upper bounding the entropy power for autoregressive processes using the estimation counterpart to Fano’s Inequality [17]

(27) $$E{\left[X-\hat{X}\left(Y_{n}\right)\right]}^{2}\geq \frac{1}{2\pi e}\exp2[h\left(X|\hat{X}\left(Y_{n}\right)\right)].$$

In our notation Equation (27) becomes

(28) $$E{\left[S-P_{n}\left(S\right)\right]}^{2}\geq \frac{1}{2\pi e}\exp2[h\left(S|P_{n}\left(S\right)\right)]$$

where *S* is the signal being predicted, $P_{n}\left(S\right)$ is the *n*th order linear predictor, and $h(S|P_{n}\left(S\right))$ is the differential entropy of the prediction error $\left[S-P_{n}\left(S\right)\right]$.

Notice that in increasing the order of an AR model, we have Equation (25), that is, the MSPE is nonincreasing with increasing model order, which then implies that

(29) $$Q_{X|P_{1}\left(X\right)}\geq Q_{X|P_{2}\left(X\right)}\ldots \geq Q_{X|P_{n-1}\left(X\right)}\geq Q_{X|P_{n}\left(X\right)}$$

holds for the sequence of entropy powers. Equation (29) follows since the iteration in Equations (19)–(25) builds the minimum mean squared error predictor as the predictor order is increased from 1 to *m*. According to Choi and Cover [29], the minimal order Gauss-Markov process that satisfies the given covariance constraints is the maximum entropy process, which implies that it is the minimum mean squared error process satisfying the constraints. Since we know that the entropy rate power $Q_{X|P_{n}\left(X\right)}$ satisfies Equation (28), Equation (29) follows.

As a setup for applications to be discussed later, note that these observations imply the following. If the AR or linear prediction coefficients and the correlation functions are known, it is straightforward to find the minimum MSPE [6,30] using Equation (24). Furthermore, if we fit an AR model to a time series, we can find the minimum MSPE from the $E^{\left(i\right)}$ generated by the recursion in Section 3 and hence upper bound the entropy rate power that corresponds to that model.

If the time series is in fact AR, we can iteratively increase the model order, calculate the AR or linear prediction coefficients for the iterated model order which yields the best fit, and for that model, we can calculate the minimum MSPE and thus an upper bound to the entropy rate power. If the time series is not AR, then we can find the best approximation and its corresponding minimum MSPE, which is again an upper bound on the entropy power for that best fit model.

## 5. Log Ratio of Entropy Powers

We can use the definition of the entropy power in Equation (3) to express the logarithm of the ratio of two entropy powers in terms of their respective differential entropies as

(30) $$\frac{1}{2}\log\frac{Q_{X}}{Q_{Y}}=\left[h\left(X\right)-h\left(Y\right)\right]$$

We are interested in studying the change in the differential entropy brought on by different signal processing operations by investigating the log ratio of entropy powers. However, in order to calculate the entropy power, we need an expression for the differential entropy! So, why do we need the entropy power?

First, entropy power may be easy to calculate in some instances, as we show later. Second, the accurate computation of the differential entropy can be quite difficult and requires considerable care [31]. Generally, the approach is to estimate the probability density function (pdf) and then use the resulting estimate of the pdf in Equation (1) and numerically evaluate the integral.

Depending on the method used to estimate the probability density, the operation requires selecting bin widths, a window, or a suitable kernel [31], all of which must be done iteratively to determine when the estimate is sufficiently accurate. The mutual information is another quantity of interest, as we shall see, and the estimate of mutual information also requires multiple steps and approximations [32,33]. These statements are particularly true when the signals are not i.i.d. and have unknown correlation.

In the following we consider special cases where Equation (30) holds with equality when the entropy powers are replaced by the corresponding variances. We then provide justification for using the signal variance rather than entropy power in other situations. The Gaussian and Laplacian distributions often appear in studies of speech processing and other signal processing applications, so we show that substituting the variances for entropy powers for these distributions satisfies Equation (30) exactly.

### 5.1. Gaussian Distributions

For two i.i.d. Gaussian distributions with zero mean and variances $\sigma_{X}^{2}$ and $\sigma_{Y}^{2}$, we have directly that $Q_{X}=\sigma_{X}^{2}$ and $Q_{Y}=\sigma_{Y}^{2}$, so

(31) $$\frac{1}{2}\log\frac{Q_{X}}{Q_{Y}}=\frac{1}{2}\log\frac{\sigma_{X}^{2}}{\sigma_{Y}^{2}}=\left[h\left(X\right)-h\left(Y\right)\right]$$

which satisfies Equation (30) exactly. Of course, since the Gaussian distribution is the basis for the definition of entropy power, this result is not surprising.

### 5.2. Laplacian Distributions

For two i.i.d. Laplacian distributions with parameters $\lambda_{X}$ and $\lambda_{Y}$ [18], their corresponding entropy powers $Q_{X}=2e\lambda_{X}^{2}/\pi$ and $Q_{Y}=2e\lambda_{Y}^{2}/\pi$, respectively, so we form

(32) $$\begin{array}{cc} \frac{1}{2}\log\frac{Q_{X}}{Q_{Y}}= & \frac{1}{2}\log\frac{\lambda_{X}^{2}}{\lambda_{Y}^{2}}\\ = & \left[\ln\left(2e\lambda_{X}\right)-\ln\left(2e\lambda_{Y}\right)\right]\\ = & [h(X)-h(Y)]. \end{array}$$

so the Laplacian distribution also satisfies Equation (30) exactly. We thus conclude that we can substitute the variance, or for zero mean Laplacian distributions, the mean squared value for the entropy power in Equation (30) and the result is the difference in differential entropies.

For speech processes, the prediction residual or prediction error often is modeled as being i.i.d. Laplacian based on speech data histograms [6,24] or simply assumed to be Gaussian for convenience [27]. As a consequence, this implies that for speech processing applications, we can use the variances of mean squared errors instead of the entropy powers without penalty, thus avoiding the calculation of the differential entropies from limited data.

Interestingly, it can be shown that the Logistic distribution [18] also satisfies Equation (30) exactly. The author has not been able to identify a broad model class for which Equation (30) is satisfied exactly.

### 5.3. Increasing Predictor Order

We can use Equation (30) in terms of the entropy powers for increasing AR model orders in the signal processing iterations as

(33) $$\frac{1}{2}\log\frac{Q_{X|P_{n-1}\left(X\right)}}{Q_{X|P_{n}\left(X\right)}}=h\left(X|P_{n-1}\left(X\right)\right)-h\left(X|P_{n}\left(X\right)\right)$$

If we add and subtract h(X) to the right hand side of Equation (33), we then obtain an expression in terms of the difference in mutual information between the two stages as

(34) $$\frac{1}{2}\log\frac{Q_{X|P_{n-1}\left(X\right)}}{Q_{X|P_{n}\left(X\right)}}=I\left(X;P_{n}\left(X\right)\right)-I\left(X;P_{n-1}\left(X\right)\right)$$

From the series of inequalities on the entropy power in Equation (29), we know that both expressions in Equations (33) and (34) are greater than or equal to zero. It is important to note that Equations (33) and (34) are not based on a Gaussian assumption for the underlying processes. For these expressions, the entropy power *Q* is calculated from the differential entropy of the signal being analyzed.

Even though the results in Equations (33) and (34) are not based on a Gaussian assumption, we know from the definition of entropy power that they hold exactly for i.i.d. Gaussian processes. Of course, because of the example in Section 5.2, we also know that these equations hold exactly for i.i.d. Laplacian processes as well. Explicitly, for two i.i.d. Laplacian distributions with parameters $\lambda_{n-1}$ and $\lambda_{n}$, their corresponding entropy powers $Q_{X|P_{n-1}}=2e\lambda_{n-1}^{2}/\pi$ and $Q_{X|P_{n}}=2e\lambda_{n}^{2}/\pi$, respectively, so we form

(35) $$\begin{array}{cc} \frac{1}{2}\log\frac{Q_{X|P_{n-1}}}{Q_{X|P_{n}}}= & \frac{1}{2}\log\frac{\lambda_{n-1}^{2}}{\lambda_{n}^{2}}\\ = & \left[\ln\left(2e\lambda_{n-1}\right)-\ln\left(2e\lambda_{n}\right)\right]\\ = & [h\left(X|P_{n-1}\right)-h\left(X|P_{n}\right)], \end{array}$$

so when the prediction error sequence at time instants n−1 and *n* satisfy, or are assumed to satisfy, the Laplacian distribution, Equation (30) is satisfied exactly. Similarly, if we add and subtract the differential entropy of the original Laplacian sequence being predicted, then Equation (34) is satisfied exactly as well. We thus conclude that we can substitute the error variance, or for zero mean Laplacian distributions, the mean squared value for the entropy power and obtain the desired relationships from the log ratio of entropy powers.

This increase in mutual information between the input and the predicted sequence also implies changes in the relationship between the prediction error sequence $X-P_{n}\left(X\right)$ and the input *X*. In fact, since we know from Equation (33) that $h\left(X|P_{n-1}\left(X\right)\right)-h\left(X|P_{n}\left(X\right)\right)\geq 0$, then we have that $h\left(X|P_{n-1}\left(X\right)\right)\geq h\left(X|P_{n}\left(X\right)\right)$. From this, we form

(36) $$h\left(X-P_{n-1}\left(X\right)|P_{n-1}\left(X\right)\right)\geq h\left(X-P_{n}\left(X\right)|P_{n}\left(X\right)\right)$$

which says that the differential entropy of the prediction error is nonincreasing with increasing prediction order. This result is consistent with the minimum mean squared prediction error (MMSPE) and entropy power results in Equations (25) and (29), and with the fact that the minimum mean squared error (MMSE) is nonincreasing with predictor order in statistical minimum mean squared error prediction [2,3], particularly for AR processes.

So, using the log ratio of entropy powers, what we have is a new interpretation of the improvement in mean squared prediction error in terms of a decrease in the differential entropy of the prediction error expressed in terms of bits. The results in terms of mutual information and differential entropy are exact for i.i.d. Gaussian and Laplacian prediction errors, but it would greatly simplify our investigations if we could infer the change in differential entropy and mutual information without checking the distributions of the data, calculating the differential entropies and/or the mutual information, and the entropy powers.

More specifically, it would be very helpful if we could substitute the mean squared prediction errors for the entropy powers in Equations (33) and (34) in general.

### 5.4. Maximum Entropy Spectral Estimate

As shown in [29], we know that the entropy of any finite length segment of a random process with a specified covariance structure is bounded above by the entropy of the minimal order Gaussian AR process that satisfies the given covariance constraints. Thus, one way to achieve the goal of using the minimum mean squared prediction error in Equations (33) and (34) for the entropy power is to assume that the linear predictor coefficients from Section 3 are the results of the given autocorrelation constraints for a maximum entropy spectrum estimate.

Since the maximum entropy spectrum is the minimal order Gauss-Markov process satisfying the given covariance constraints, the minimum mean squared prediction errors equal the entropy powers and therefore can be used in Equations (33) and (34) [29], thus avoiding the need to calculate the entropy power directly using Equation (3).

Following [29], by assuming the maximum entropy estimate of the spectral density, we are not assuming that the higher order, k>m, covariance terms in Equation (10) are equal to zero but that they satisfy

(37) $$\phi \left(k\right)=\sum_{j=1}^{m}a_{j}^{\left(m\right)}\phi \left(k-j\right)$$

for k≥m+1. However, for our development, this fact is unimportant.

### 5.5. Orthogonal Decompositions and Whitened Prediction Errors

We can also motivate using the minimum mean squared prediction error for the entropy power in Equations (33) and (34) by considering the series of AR predictors obtained in Section 3 as *n* progresses from 1 to *m* as a series of orthogonal decompositions into deterministic and nondeterministic processes [2,3,34], producing a whitened prediction error at each stage. Of course, this idea is not far-fetched because it is intuitive that the goal of the linear predictor for each *n* is to obtain the whitest prediction error possible [27].

Conceptually, this is equivalent to assuming that for each *n*, the process is exactly an nth order AR process. Under these assumptions, we would have a series of minimum mean squared prediction errors expressible as [2,3]

(38) $$E^{\left(n\right)}=\exp[\frac{1}{2\pi }\int_{-\pi }^{\pi }\log\Phi_{AR(n)}\left(\omega \right)d\omega ]$$

n=1,2,…,m, where $\Phi_{AR(n)}\left(\omega \right)$ is the power spectral density of the corresponding nth order AR process, which holds even if the AR processes are non-Gaussian. Since the mean squared prediction error $E^{\left(n\right)}$ is exactly the entropy power if the series of nth order AR processes are Gaussian (by definition of entropy power), it is natural to use the minimum mean squared prediction error for the entropy power under these conditions.

In summary, based on (a) the fact that Equation (30) is satisfied exactly for i.i.d. Gaussian and Laplacian random variables, as can be seen from Equations (31) and (32), respectively; (b) the fact that the maximum entropy spectral estimate for the given correlation constraints is a Gaussian AR process; and (c) the fact that the linear predictors that yield the best whitened prediction errors for each order produce a minimum mean squared prediction error that satisfies Equation (38), which is the expression for the entropy power for a Gaussian AR process, we proceed in the following to replace the entropy power in key equations, such as Equations (33) and (34) with the minimum mean squared prediction error.

To reiterate, the purpose of using the minimum mean squared prediction error, which is readily available, is to eliminate the need to numerically calculate/estimate the histogram of the data and then use the histogram to form the entropy power from Equation (3). This is a substantial simplification that is very useful in practice and also is a process that eliminates the histogram estimation step, which as discussed earlier in this section, is in itself fraught with sources of inaccuracies.

Examples and applications of the log ratio of entropy powers are developed in the following sections.

## 6. Experimental Examples

To illustrate the utility of the expression in Equation (34), we consider four frames of narrowband voiced speech. For each frame, we list the MMSPE as the predictor order is incremented up to order N=10 and also list the corresponding change in mutual information as calculated from Equation (34) as the model order is increased, but where we use $MMSPE\left(X,X_{n}\right)=E^{\left(n\right)}$, as in Equations (24) and (25) in place of $Q_{X|P_{n}\left(X\right)}$.

To categorize the differences in the speech frames we investigate, we use a common indicator of predictor performance, the Signal-to-Prediction Error (SPER) in dB [35], also called the Prediction Gain [6], defined as

(39) $$SPER\left(dB\right)=10\log_{10}\frac{MSE(X)}{MMSPE(X,X_{10})}$$

where MSE(X) is the average energy in the utterance and $MMSPE(X,X_{10})$ is the minimum mean squared prediction error achieved by a 10th order predictor. The SPER can be calculated for any predictor order but we choose N=10, a common choice in narrowband speech codecs and the predictor order that, on average, captures most of the possible reduction in mean squared prediction error without including long term pitch prediction.

**Figure 1:**
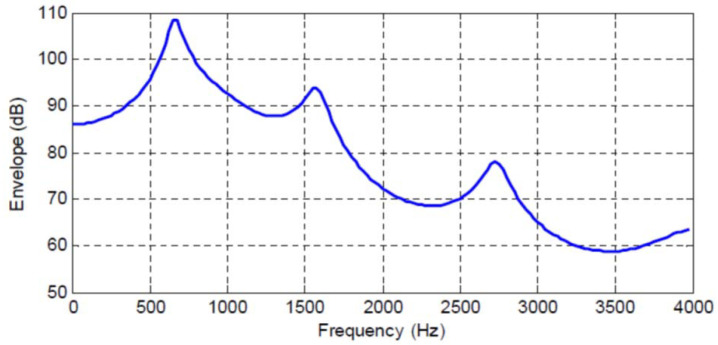
Frame 45 Spectral Envelope, SPER = 16 dB.

**Figure 2:**
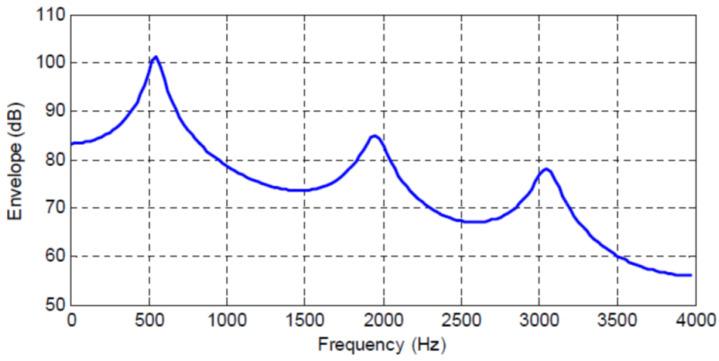
Frame 23 Spectral Envelope, SPER = 11.5 dB.

**Figure 3:**
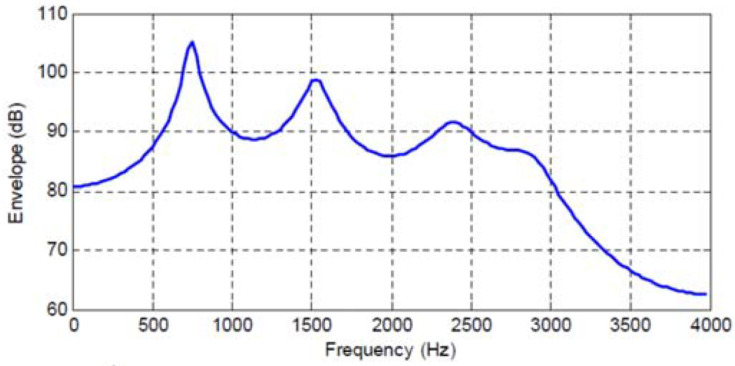
Frame 3314 Spectral Envelope, SPER = 7.74 dB.

**Figure 4:**
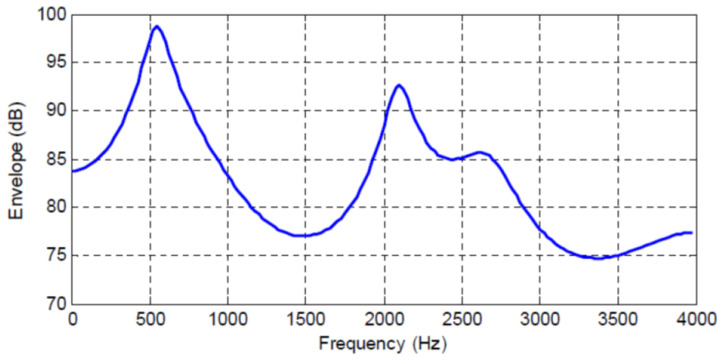
Frame 87 Spectral Envelope, SPER = 5 dB.

Figure 1, Figure 2, Figure 3 and Figure 4 show the spectral envelopes calculated using an N=10th order AR model for four different speech frames. Correspondingly, Table 1, Table 2 and Table 3 list the $MMSPE\left(X,X_{N}\right)=E^{\left(N\right)}$ as the predictor order is increased from N=1 up to N=10 in increments of one for the speech frames in Figure 1, Figure 2 and Figure 3. We do not include a table or Frame 87 since the MMSPE is only reduced from MSE(X)=1.0 to $MMSPE(X,X_{10})=0.316$. The third column in the tables labeled as $I\left(X;X_{N}\right)-I\left(X;X_{N-1}\right)$ is obtained from Equation (34) but with the entropy power for each *N* replaced by the $MMSPE(X,X_{N})$ so we have

(40) $$\frac{1}{2}\log\frac{MMSPE(X,X_{N-1})}{MMSPE(X,X_{N})}=I\left(X;X_{N}\right)-I\left(X;X_{N-1}\right)$$

In this equation and the tables, we interpret the mutual information $I(X;X_{n})$ when n=0 as equal to zero since in that case $X_{n}=0$.

We are approximating the entropy power $Q_{X|P_{N}\left(X\right)}$ by $MMSPE(X,X_{N})$ at each stage, since the latter is much simpler to calculate. While this can be argued to be equivalent to assuming that the speech signal is Gaussian, thus leading to Equations (27) and (28) being satisfied with equality, we are not arguing that speech signals have a Gaussian distribution. Our goal instead is to characterize the incremental change in mean squared prediction error for AR processes in terms of gains in mutual information. The results are interesting and potentially highly useful in many applications.

The four speech frames being considered have quite different SPER values, specifically SPERs = 16, 11.85, 7.74 and 5 dB, and thus constitute an interesting cross section of speech segments to be analyzed; however, although different, the average SPER of these four frames is about 10 dB, which is the usual SPER or prediction gain that is shown for average speech data and N=10 in the literature [6,24]. The first significant observation is that now we can map an increase in predictor order to an increase in the mutual information between the input sequence and the Nth order predicted value, which is an entirely different interpretation and a new indicator of predictor quality.

In particular, we see that the N=10th order predictors corresponding to the spectral envelopes in Figure 1, Figure 2, Figure 3 and Figure 4 increase the mutual information between the input segment and predicted signal by 2.647, 1.968, 1.29, and 0.83 bits/sample, respectively. We also see the incremental change in mutual information as the predictor order is indexed up from 1 to *N*. This characterization opens up new analysis and research directions by extending the MSE to an information measure.

Along these lines of analysis, note that in Table 1 when the predictor order is increased from N=1 to N=2, the mean squared error changes by 0.311 − 0.066 = 0.245, which as shown in the table corresponds to an increase in mutual information between the original input and the predicted value of 1.11 bits/letter. While the normalized change in mean squared error is noticeable, the increase in mutual information is more than 1 bit! For the same table, we see that in increasing the predictor order from N=3 to N=4 the mean squared error changes by 0.0587 − 0.0385 = 0.0202, a seemingly very small change, but from Table 1 we see that the corresponding increase in mutual information is 0.304 bits/letter. Thus, the change in mutual information captures more clearly an improvement perhaps not as evident in the change in mean squared error. This behavior is also evident in the other tables.

For example, in Table 2 when the predictor order is increased from N=1 to N=2 the mean squared error changes by 0.096 while the increase in mutual information is 0.339 bits/letter and similarly as the predictor order is increased from N=6 to N=7 the mean squared error changes by 0.0258 while the increase in mutual information is 0.226 bits/letter. We can see other such occurrences in Table 3 when the predictor order is increased from N=5 to N=6, the mean squared error changes by 0.1365 while the increase in mutual informaton is 0.36 bits/letter, and as the predictor order is increased from N=7 to N=8, the mean squared error changes by 0.0277 while the increase in mutual information is 0.104 bits/letter. It is therefore clear, that the log ratio of entropy powers provides significantly different, but complementary, insights to the observed changes in mean squared prediction error.

For some applications, such as speech waveform coding, the interpretation is direct in terms of the decrease in average number of bits/sample required for transmitting or storing the waveform, as developed in Section 7.1. More generally, the incorporation of an information measure into analyses of seismic, speech, EEG, and ECG signals offers a new way forward for researchers modeling these signals for analysis, detection, and classification. The change in mutual information as the linear predictor order is adjusted can also provide a new interpretation and categoration of the codebooks used in code-excited linear predictive speech coding, which is discussed in Section 7.2. In any event, these results suggest an interesting new, and previously unexplored way for researchers to classify the contributions of AR predictors and linear prediction in their applications.

## 7. Application to Speech Coding

One particularly interesting and useful application is to linear prediction-based speech coding, which constitutes the primary underlying principle for most deployed speech codecs today for digital cellular and Voice over Internet Protocol (VoIP) [36,37,38]. More specifically, Equation (40) may be useful in characterizing the bit rate reduction for speech coding produced by increasing the linear (AR) predictor order.

### 7.1. Speech Waveform Coding

For linear prediction-based time-domain waveform coding such as differential pulse code modulation (DPCM), a common performance indicator is the signal to noise ratio (SNR) defined as

(41) $$SNR=\frac{MSE(X)}{MSE(X,X_{R})}$$

where $X_{R}$ is the reconstructed sequence at the decoder and thus $MSE(X,X_{R})$ is the mean squared reconstruction error [16,35]. A characterization of the performance in terms of the achieved SNR has always been in terms of the SPER, sometimes also called the Prediction Gain [6,36] from the expression

(42) $$SNR=SPER\cdot SNR_{Q}$$

where SPER=1/MMSPE and $SNR_{Q}$ is the signal-to-noise ratio of the quantizer [6], or equivalently

(43) $$SNR\left(dB\right)=SPER\left(dB\right)+SNR_{Q}\left(dB\right)$$

where $SNR\left(dB\right)=10\log_{10}SNR$. In these equations, we see that the SNR has an association with bit rate through the number of levels or output entropy of the quantizer; however, the SPER is only associated with the error in prediction and only indirectly with the reconstruction error versus bit rate. A natural question that follows is “How many bits/sample does an SPER or prediction gain of a certain magnitude save when coding the speech?” Equation (40) allows us to estimate this savings in bit rate.

To elaborate, suppose that we wish to compare the performance of 8 bits/sample μ-law PCM [16,35] that achieves a desirable target SNR of SNR(dB)=35dB with the performance of adaptive DPCM, particularly with the goal of determining how much prediction gain is required. While we can investigate the bits/sample required through Equations (42) and (43) indirectly, our new results allow us to write a new additive bit rate equation of the form

(44) $$R_{SNR}=R_{SPER}+R_{SNR_{Q}}.$$

This result says that the rate $R_{SNR}$ that produces a desired overall SNR in PCM can be expressed as the summation of the effective rate compensated for by the linear prediction, $R_{SPER}$, and the rate of the quantizer, $R_{SNR_{Q}}$, needed to produce the desired output SNR when operating on the prediction error. Although simple, this is a fascinating result. The distortion-rate performance of the ADPCM system is broken down into components of the SNR due to prediction and to quantization as in Equations (42) and (43) and the effective rate of each of these components as in Equation (44). A few examples of the utility of this new characterization follow.

As a concrete example from Figure 1 and Table 1, we see that an N=10th order predictor allows a reduction in bit rate of 2.647 bits/sample over direct pulse code modulation (PCM), that is, coding of the original speech signal without prediction. Taking 8 bits/sample μ-law PCM as our reference and using Equation (44), this implies that using predictive coding, the bit rate would be reduced to about 5.4 bits/sample for the prediction error quantization, or 43.2 kbits/s. To factor in the perceptual difference between log-PCM quantization noise and the quantization noise in predictive differential encoding, we can use some earlier results on such a comparison between 7 bits/sample μ-law PCM and ADPCM that aligns the objective SNR with subjective ratings by listeners to see that a 2.647 bits/sample reduction from 7 bits/sample μ-law PCM would yield a required bit rate of 4.4 bits/sample or 35.2 kbits/s, which closely fits the subjective performance results in [6,39] and the performance achieved by 4 bits/sample or 32 kbits/ ADPCM in the G.726 standard [40].

We can also observe from Table 1 that well over half of the total gain in bit rate for an N=10th order predictor is obtained with only an N=4th order predictor which achieves a rate reduction of 2.348 bits/sample. While it is evident that an N=4th order predictor for Frame 45 accomplishes most of the reduction in MSPE, casting the performance of an N=4th order predictor *as a reduction in bit rate* of more than 2.25 bits/sample seems to capture more explicitly the importance of the predictor for representing this frame of speech.

If we consider a similar analysis for the three other speech frames in Figure 2, Figure 3 and Figure 4, we see that these frames have a lower SPER than for Frame 45, and that from Equation (44) in order to maintain a constant $R_{SNR}$, the rate of the quantizer must be higher as the bit rate reduction provided by the predictor is 1.968, 1.29, and 0.83 bits/sample, respectively, for these three frames. While it is known and intuitive that poorer predictor performance must be compensated for by the quantizer, this explicit characterization in terms of rate is informative and new.

### 7.2. Code-Excited Linear Prediction

**Figure 5:**
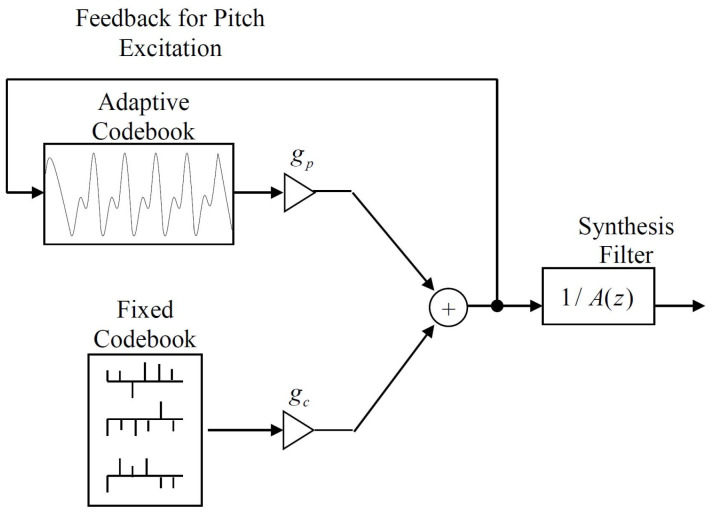
Code-Excited Linear Prediction (CELP) Decoder.

For speech coding, we can go beyond linear predictive waveform coding and consider the utility of our results to Code-Excited Linear Prediction (CELP), which does not attempt waveform-following, but uses linear prediction for the short term memory along with a fixed codebook excitation and an adaptive codebook, the last of which captures the long term memory due to speaker pitch. A block diagram of a CELP decoder is shown in Figure 5. In this figure, $A\left(z\right)=1-\sum_{j=1}^{m}a_{j}z^{-j}$ is the *z* domain representation of the AR model in Equation (13) and $g_{p}$ and $g_{c}$ are the gains associated with the adaptive codebook and the fixed codebook, respectively. The predictor coefficients $a_{j},j=1,2,\ldots ,m$, the codebook gains $g_{p}$ and $g_{c}$, the adaptive codebook long term memory or speaker pitch estimate, and the specific fixed codebook codeword are selected at the CELP Encoder (not shown) to provide the best frequency weighted squared error fit over the current speech frame and then transmitted to the decoder for speech synthesis. For more details on the CELP structure and the encoding and decoding processes, the reader should consult the references [6,16,24,36].

CELP is the most widely deployed method for speech coding today, serving as the primary speech coding method in the Adaptive Multirate (AMR) codec [37] and in the Enhanced Voice Services (EVS) codec [38] used in cell phones and VoIP. Interestingly, although signal-to-noise ratio is not a useful overall performance indicator for CELP codecs, we develop the idea that the MMSPE for the AR modeling of the short-term memory can give a useful measure for the reduction, or increase, in the bits or complexity needed for the codec excitation.

We consider the case where a CELP codec is used to code the AR sequence corresponding to Figure 1, Figure 2, Figure 3 and Figure 4. Since it is well known that the fixed and adaptive codebook excitation must make up for any error energy not modeled by the short term predictor [36], in applying the results in Table 1 based on Equation (40) for CELP, the change in bits/sample provided by an increase in linear predictor order can be interpreted as a corresponding change (reduction) in the bits/sample, or codebook complexity, required by the fixed codebook.

To make this more specific, let the combined Fixed and Adaptive Codebook excitation for the Synthesis Filter in Figure 5 be denoted as $X_{C}$ and the reconstructed output as $X_{R}$, and then we form

(45) $$I\left(X;X_{R}\right)=I\left(X;X_{N},X_{C}\right)=I\left(X;X_{N}\right)+I\left(X;X_{C}|X_{N}\right)$$

where $X_{N}$ is the prediction term and *X* is the original speech being coded by the using the CELP Encoder (not depicted). This expression states that the mutual information between the original speech *X* and the reconstructed speech $X_{R}$ equals the mutual information between *X* and $X_{N}$, the linear prediction of *X*, plus the mutual information between *X* and the codebook excitations $X_{C}$ conditioned on $X_{N}$. Thus, to achieve or maintain a specified mutual information between the original speech and the reconstructed speech, any change in $I(X;X_{N})$ must be offset by an adjustment of $I(X;X_{C}|X_{N})$.

We note from Figure 5 that the codebook excitations consists of two additive components, the Adaptive codebook and the Fixed codebook. We thus can decompose the term $I(X;X_{C}|X_{N})$ as

(46) $$\begin{array}{cc} I(X;X_{C}|X_{N})= & I(X;X_{A},X_{F}|X_{N})\\ = & I\left(X;X_{A}|X_{N}\right)+I\left(X;X_{F}|X_{N},X_{A}\right)\\ = & I\left(X;X_{F}|X_{N}\right)+I\left(X;X_{A}|X_{N},X_{F}\right) \end{array}$$

where $X_{A}$ represents the Adaptive codebook contribution and $X_{F}$ represents the Fixed codebook contribution. Both forms of the chain rule for the mutual information are shown in Equation (46), but in most codec designs, the adaptive codebook contribution is removed before the search over the fixed codebook.

For our current purposes, however, the term of interest is $I(X;X_{N})$, which we suggest can be calculated/approximated by the log ratio of MMSPE values as in Table 1, Table 2 and Table 3, and in Figure 4. Therefore, we avoid the difficult calculations and approximations needed to estimate the differential entropies and the mutual information, and thus we can more easily get insights into the tradeoffs between the prediction component and the codebook excitations through Equations (45) and (46) for CELP codec designs.

## 8. Other Possible Applications

Of course, AR models play a large role in many applications, including ECG classification [9], EEG classification [10,11], and geophysical exploration [7,8]. We have not conducted experiments using our log ratio of entropy powers approach to these problems, but, in the following subsections, we outline in some detail possible approaches to a few of these applications. The applications described are chosen such that each application uses the AR model in different ways. Sufficient details are provided such that researchers skilled in these several areas should be able to follow the descriptions to implement their particular application.

### 8.1. ECG Classification

Reference [9] describes a method for electorcardiogram (ECG) classification and detection based on AR models of the QRS complex in an ECG signal. The overall method consists of multiple signal processing steps, beginning with preprocessing to remove artifacts from respiratory modulation and baseline drift, then filter bank methods to identify QRS peaks, followed by AR modeling of one or two beats of the ECG signal available from the pre-processing step, and finally clustering to arrive at a classification or patient identification.

The classification task is to determine whether an ECG shows a normal heart rhythm, an arrhythmia, or a ventricular arrhythmia. The patient identification task is to recognize a patient from a recorded ECG. The AR modeling step is the central component of the method, and the authors state that having an accurate AR model order is crucial to the success of both tasks and that using the prediction error power in addition to the predictor coefficients significantly improves the patient identification task. Of course, both the prediction error power and the model order are intimately intertwined, since the former is an output of the latter.

The authors in [9] determine the model order from the observation of a “knee” in the modeling error power curve as the model order is increased. In their work, they use orders of 2–4, which do, in fact, yield a small MSPE for the one example specifically presented. We suggest that the log ratio of entropy powers as the model order is increased as presented in Section 5.3 of the current paper can serve as an additional indicator of a suitable AR model order through Equations (33) and (34) with the entropy powers replaced by MSPE as in Equation (40).

Specifically, from the examples in Section 6, we have seen that the log ratio of entropy powers can be interpreted as a possibly more sensitive indicator of the match produced as the model order is increased in comparison to the straightforward MSPE. Since for the patient identification task, the prediction error power improved recognition performance, folding in the change in mutual information as from Equation (40) may supply a further improvement in performance.

### 8.2. EEG Classification

Autoregressive models have been used in a variety of EEG signal processing applications. One such application is the classification of an EEG to determine if a patient has sufficient anethesia for deep surgery [10]. The approach in [10] is to develop m=1,2,…,MNth order AR models, denoted as $AR^{\left(m\right)}$ from EEG signals taken from other patients that are labeled as being in some state (inadequate anethesia, sufficient anethesia), which are then used as templates representing the different states of anethesia. The challenge is that while the template EEGs are labeled as being in one of two classes, the interpatient variability is high, so a statistical comparison of the input EEG is needed to see which template it is closest to.

To accomplish this, the input EEG is passed through the prediction error filters formed from all of the $AR^{\left(m\right)}$m=1,2,…,MNth order AR models, with the output of each filter producing a prediction error that is the result of predicting the input EEG with the AR coefficients determined from each labeled template. Each template covariance matrix and its’ corresponding MMSPE are fed to a Gaussian assumption Karhunen-Loeve Nearest Neighbor (KL-NN) rule to select the correct classification.

In [10,11] the order of all of the $AR^{\left(m\right)}$ models are set at some fixed *N*, and it is possible that the change in mutual information as indicated by the log ratio of entropy powers could be used to aid in the selection of the appropriate model order. However, as an alternative application, we suggest here that the log ratio of entropy power between the EEG being tested and each of the template $AR^{\left(m\right)}$ models might be a good classification rule.

What we propose is to use the prediction errors at the output of each prediction filter as in reference [10] and determine the MMSPE for each, then form the log ratio of entropy powers to compare each of these to the MMSPE for each template, and then use these quantities in a NN algorithm. Notationally, let the MMSPE for each template $AR^{\left(m\right)}$ be $Q(X_{m}|AR^{\left(m\right)})$, m=1,2,…,M, where $X_{m}$ is the EEG used to develop template *m*, and let the MMSPE when predicting the input EEG with each $AR^{\left(m\right)}$ be $Q(Y|AR^{\left(m\right)})$, where *Y* denotes the input EEG signal. By the definition of the optimality of the templates in being matched to their particular mth EEG signal, we know that

(47) $$Q\left(Y|AR^{\left(m\right)}\right)\geq Q(X_{m}|AR^{\left(m\right)})$$

for m=1,2,…,M.

We can thus form the log ratio of entropy powers as

(48) $$\begin{array}{cc} d(Y,X\left(AR^{\left(m\right)}\right))= & \frac{1}{2}\log\frac{Q(Y|AR^{\left(m\right)})}{Q(X_{m}|AR^{\left(m\right)})}\\ = & h\left(Y|AR^{\left(m\right)}\right)-h\left(X|AR^{\left(m\right)}\right) \end{array}$$

which we know is greater than or equal to zero. A mutual information expression for the log ratio of entropy powers does not appear to be possible for this problem setup. The quantity in Equation (48), $d(Y,X\left(AR^{\left(m\right)}\right))$, can then be used directly in a NN calculation as in [10].

### 8.3. Geophysical Exploration

There is a very long history of autoregressive or linear prediction models in geophysical signal processing and exploration [7,8,41,42] for use in developing an understanding of the subsurface crustal layer interfaces. In those applications, the predictor order *N* may be chosen to be (1) some previously determined length, or (2) the predictor order where the normalized mean squared error meets some prior-selected threshold or shows only slight further increases.

From Table 1, Table 2 and Table 3, we see the normalized MMSPE as the predictor order is increased from N=1 to N=10. Observing these values, it would appear that choosing a preset value for the predictor order in this range would not produce reliable results, except perhaps if *N* were chosen to be large. Additionally, selecting a predetermined threshold for the MMSPE to select the predictor order also appears to be difficult. However, selecting the predictor order where the normalized mean squared prediction error shows only slight further increases as *N* is incremented appears workable. Of course, quantifying “only slight further increases” can be a challenge.

As discussed in detail toward the end of Section 6, the mutual information or the log ratio of entopy powers provides an alternative indicator of the new “information” being obtained as the predictor order is incremented. Reiterating a few of those points, we see that in Table 1 when the predictor order is increased from N=3 to N=4 the mean squared error changes by 0.0202, an apparently small change, but the corresponding increase in mutual information is 0.304 bits/letter. Similarly, in Table 2 when the predictor order is increased from N=6 to N=7 the mean squared error changes by 0.0258, less than three hundredths of the normalized energy, while the increase in mutual information is 0.226 bits/letter. Additionally, from Table 3 when the predictor order is increased from N=7 to N=8, the mean squared error changes by 0.0277, a relatively small value, while the increase in mutual information is 0.104 bits/letter.

Therefore, for the geophysical application of the AR model, the log ratio of entropy powers or change in mutual information provides a perhaps more sensitive indicator of the appropriate model order and certainly a new intuitive interpretation of changes in mean squared prediction error as the model order is increased.

## 9. Conclusions

We present a new quantity, the log ratio of entropy powers, for investigating the changes in mutual information and differential entropy as the predictor order is incremented in autoregressive models or for evaluating the overall change in differential entropy and mutual information for a selected AR model order. We show that entropy power can be replaced by the minimum mean squared prediction error and the expressions still hold for i.i.d. Gaussian and Laplacian signals, Gaussian autoregressive processes, maximum entropy spectral estimates, and AR decompositions into deterministic and nondeterministic components. We then use the log ratio of entropy power expression with MMSPEs substituted for the entropy powers and study AR modeling of speech and applications to speech coding analysis. The analyses allow us to associate a specific performance gain in terms of bits/sample with the reduction in MSPE provided by the predictor for speech waveform coding. For CELP coding the overall reduction in MSPE can be interpreted in terms of a change in mutual information, which allows us to decompose the mutual information between the input speech being coded and the reconstructed speech into a sum of mutual informations and conditional mutual informations provided by the codec components. We also develop approaches to apply the new quantity to the AR models used for seismic exploration, EEG classification, and ECG classification. We feel that the log ratio of entropy powers is an insightful new tool for the analysis of AR models and for the analysis of AR models used in many different types of applications.

## Figures and Tables

**Table 1:** Change in Mutual Information from Equation (34) as the Predictor Order is Increased: Frame 45, SPER = 16 dB.

*N*	$\mathrm{MMSPE}(X,X_{N})$	$I\left(X;X_{N}\right)-I\left(X;X_{N-1}\right)$
0	1.0	0 bits/letter
0–1	0.3111	0.842 bits/letter
1–2	0.0667	1.11 bits/letter
2–3	0.0587	0.092 bits/letter
3–4	0.0385	0.304 bits/letter
4–5	0.0375	0.019 bits/letter
5–6	0.0342	0.065 bits/letter
6–7	0.0308	0.069 bits/letter
7–8	0.0308	0.0 bits/letter
8–9	0.0261	0.12 bits/letter
9–10	0.0251	0.026 bits/letter
0–10 Total	0.0251	2.647 bits/letter

**Table 2:** Change in Mutual Information from Equation (34) as the Predictor Order is Increased: Frame 23, SPER = 11.85 dB.

*N*	$\mathrm{MMSPE}(X,X_{N})$	$I\left(X;X_{N}\right)-I\left(X;X_{N-1}\right)$
0	1.0	0 bits/letter
0–1	0.2577	0.978 bits/letter
1–2	0.1615	0.339 bits/letter
2–3	0.1611	0.0 bits/letter
3–4	0.1179	0.225 bits/letter
4–5	0.1118	0.042 bits/letter
5–6	0.0962	0.104 bits/letter
6–7	0.0704	0.226 bits/letter
7–8	0.0653	0.054 bits/letter
8–9	0.0653	0.0 bits/letter
9–10	0.0652	0.0 bits/letter
0–10 Total	0.0652	1.968 bits/letter

**Table 3:** Change in Mutual Information from Equation (34) as the Predictor Order is Increased: Frame 3314, SPER = 7.74 dB.

*N*	$\mathrm{MMSPE}(X,X_{N})$	$I\left(X;X_{N}\right)-I\left(X;X_{N-1}\right)$
0	1.0	0 bits/letter
0–1	0.6932	0.265 bits/letter
1–2	0.4918	0.25 bits/letter
2–3	0.4782	0.02 bits/letter
3–4	0.3554	0.215 bits/letter
4–5	0.3474	0.0164 bits/letter
5–6	0.2109	0.36 bits/letter
6–7	0.2065	0.015 bits/letter
7–8	0.1788	0.104 bits/letter
8–9	0.1682	0.044 bits/letter
9–10	0.1680	0.0 bits/letter
0–10 Total	0.1680	1.29 bits/letter

## Abbreviations

The following abbreviations are used in this manuscript:

ADPCM	Adaptive differential pulse code modulation
AMR	Adaptive multirate
AR	Autoregressive
CELP	Code-excited linear prediction
DPCM	Differential pulse code modulation
ECG	Electorcardiogram
EEG	Electroencephalogram
EVS	Enhanced voice services
MSPE	Mean squared prediction error
MMSPE	Minimum mean squared prediction error
MMSE	Minimum mean squared error
PCM	Pulse code modulation
SNR	Signal to noise ratio
SPER	Signal to prediction error ratio
VoIP	Voice over Internet Protocol
